# Exploring how changes to the steroidal core alter oleogelation capability in sterol: γ‐oryzanol blends

**DOI:** 10.1002/aocs.12624

**Published:** 2022-07-29

**Authors:** Andrew B. Matheson, Georgios Dalkas, Gareth O. Lloyd, Aaliyah Hart, Arjen Bot, Ruud den Adel, Vasileios Koutsos, Paul S. Clegg, Stephen R. Euston

**Affiliations:** ^1^ School of Physics and Astronomy University of Edinburgh Edinburgh UK; ^2^ School of Engineering and Physical Sciences, Institute of Biological Chemistry, Biophysics and Bioengineering Heriot‐Watt University Edinburgh UK; ^3^ School of Chemistry, Joseph Banks Laboratories University of Lincoln Lincoln UK; ^4^ Unilever Foods Innovation Centre Wageningen The Netherlands; ^5^ Laboratory of Physics and Physical Chemistry of Foods, Department of Agrotechnology and Food Sciences Wageningen University and Research Wageningen The Netherlands; ^6^ School of Engineering, Institute for Materials and Processes The University of Edinburgh Edinburgh UK

**Keywords:** fat substitutes, nutraceuticals/functional foods, rheology, structure–functional properties

## Abstract

Oleogels based on sterols such as β‐sitosterol blended with the sterol ester γ‐oryzanol are a very interesting class of systems, but there are aspects of their formation and structure that remain elusive. It has previously been shown that a methyl group on the C30 position of the sterol‐ester plays an important role in gelation. This work explored the effect that having C30 methyl groups on both the sterol and the sterol‐ester had on the gelation process and subsequent gel structure. Lanosterol and saponified γ‐oryzanol (which was synthesized as part of this study) were identified as materials of interest, as both feature a methyl group on the C30 position of their steroidal cores. It was observed that both sterols formed gels when blended with γ‐oryzanol, and also that lanosterol gelled sunflower oil without the addition of γ‐oryzanol. All of these gels were significantly weaker than that formed by β‐sitosterol blended with γ‐oryzanol. To explore why, molecular docking simulations along with AFM and SAXS were used to examine these gels on a broad range of length scales. The results suggest that saponified γ‐oryzanol‐γ‐oryzanol gels have a very similar structure to that of β‐sitosterol‐γ‐oryzanol gels. Lanosterol‐γ‐oryzanol gels and pure lanosterol gel, however, form with a totally different structure facilitated by the head‐to‐tail stacking motif exhibited by lanosterol. These results give further evidence that relatively slight changes to the molecular structure of gelators can result in significant differences in subsequent gel properties.

## INTRODUCTION

**FIGURE 1:**
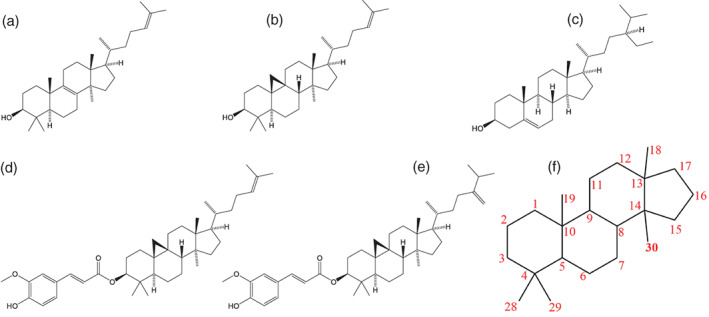
Chemical structures of (a) lanosterol, (b) cycloartenol, (c) β‐sitosterol, (d) cycloartenyl ferulate, and (e) 24‐methylenecycloartanyl ferulate (the major components of γ‐oryzanol). (f) Is a diagram showing the numbering system for steroidal cores, with the 30 methyl group highlighted. 20–27 are associated with the acyclic tail and have been omitted for clarity.

Oleogels are systems where a network formed from oleogelating structurants entraps liquid oil, resulting in the formation of a soft‐solid. These have numerous potential applications in food, pharmaceuticals, and petrochemicals. One of the most widely studied gels is that formed by blends of the sterol β‐sitosterol and the sterol‐ester γ‐oryzanol (Matheson, Dalkas, Clegg, et al., 2018; Poole et al., 2020). These gels are formed when β‐sitosterol and γ‐oryzanol associate into hollow tubules of ~10 nm in diameter (Bot et al., 2012; Dalkas et al., 2018; Matheson et al., 2017; Matheson, Dalkas, Mears, et al., 2018). Although previous studies have shown steranes (which lack a hydroxyl group, such as cholestane) will not gel with γ‐oryzanol (Bot & Agterof, 2006), to date no sterol has been found which will not. Along with spectroscopy results and molecular docking simulations, this supports the hypothesis that intermolecular hydrogen bonding between the hydroxyl group on the sterol and carbonyl group on the ester is a prerequisite to self‐assembly (den Adel et al., 2010; Pernetti et al., 2007). Initially, it was thought that intermolecular hydrogen bonding alone was key to the formation of these tubules, but recently it has been shown that a more complex balance of hydrogen bonding and van der Waals forces is responsible and that slight changes to the structure of the steroidal core of γ‐oryzanol may be enough to prevent self‐assembly with β‐sitosterol. Specifically, the methyl group at the C30 position of the cycloartenyl core was identified as being of key importance in ensuring the steroidal cores of the sterol and sterol‐ester sit on top of each other with the correct orientation (Dalkas et al., 2018). Sterols other than β‐sitosterol (such as cholestanol, stigmasterol, and cholesterol) interact with γ‐oryzanol, and it has been observed that they may form gels when blended with γ‐oryzanol. Although the steroidal cores of each of these molecules differed, none has a methyl group on the C30 position of their steroidal core. Thus although the presence of the C30 methyl group on the γ‐oryzanol molecule is clearly important, the importance of the *absence* of this group from the β‐sitosterol molecule in gel formation has not been explored. To that end, this work explored whether a blend of a sterol and sterol ester in which *both* molecules had a methyl group at 30 would form tubules and thus gels in the same manner. Two possible materials were identified to test this—lanosterol (which is commercially available), and the sterols produced through the saponification of γ‐oryzanol (the major components of which should be cycloartenol and 24‐methylene cycloartenol) which were synthesized as part of this study. The structures of these materials are shown in Figure 1.

## METHODS

### Materials and synthesis

Reagents were purchased from Merck Aldrich, except for β‐sitosterol and γ‐oryzanol, which were supplied by Unilever. Previous analysis gave the composition of the γ‐oryzanol as—45.9% 24‐methylene cycloartenyl ferulate, 26.8% cycloartenyl ferulate, 13.1% campesteryl ferulate, 7.1% sitosteryl ferulate, 1.4% D5‐avenasteryl ferulate, 1.3% stigmasteryl ferulate, 1.0% campestanyl ferulate, and the composition of the β‐sitosterol as—78.5% β‐sitosterol, 10.3% β‐sitostanol, 8.7% campesterol, 0.9% campestanol (Bot et al., 2008). Saponification of γ‐oryzanol was performed in several solvent and basic salts combinations. Tetrahydrofuran or methanol: water and KOH did not work effectively, with ethanol:water (8:2) 200 ml and NaOH (four equivalents) of 2 g of γ‐oryzanol refluxed over 4 days producing quantitative yields of the saponified γ‐oryzanol. After the yellow solution was neutralized with HCl, separation was performed using 3 × 100 ml CHCl_3_. The organic phases were combined, dried using anhydrous magnesium sulfate, and the solvent removed under reduced pressure on a rotavap. No further purification was performed. Lanosterol was purchased from Abcam (ab 143883) with purity >55%.

Gels were prepared by weighing out gelator materials (either as pure samples or 1:1 molecular ratio of sterol to γ‐oryzanol), then stirring in sunflower oil heated to ~90°C until all materials had dissolved. Stock samples were then stored at room temperature, and reapportioned and (if necessary) melted again as required for further measurements.

### Experimental

Rheology was carried out using a TA instruments AR2000 rheometer equipped with 4 cm cross‐hatched plates, and with a gap set to ~1 mm. Molten gels were poured onto the rheometer bottom plate and an oscillatory stress of 10 Pa at 1 Hz was immediately applied, this being a high enough stress to encourage gelation but not so strong as to cause the nascent gels to yield (Bot & Agterof, 2006). The bottom plate of the rheometer was maintained at approximately 20°C. All rheology measurements were replicated at least once, and qualitatively the same behavior observed.

Atomic force microscopy (AFM) was carried out using a Bruker Multimode/Nanoscope IIIa (Bruker, Santa Barbara, CA) atomic force microscope operating in tapping mode. The instrument was equipped with a J‐scanner (lateral scan range of ∼140 μm), Bruker cantilevers (model MPP‐11220‐10) with nominal spring constant of 40 N/m and resonant frequency of 300 kHz, and tips with nominal tip radius of 8 nm. For AFM, samples were prepared in the manner described in Matheson et al. (2017), which was in turn based on the method developed by Sawalha et al. for SEM samples (Sawalha et al., 2015). Briefly, samples are dipcast onto mica substrates and then left to set overnight quiescently in ambient room temperature (~20°C). A few hours before measurements, samples were then dipped in ethanol to remove any small amounts of free oil which may be on the sample surface. This allowed images to be obtained without fully drying the sample of all oil, thus preserving the microstructure. Images were post‐processed and analyzed using the built in tools of the Gwyddion software package (Nečas & Klapetek, 2012), firstly a polynomial background is subtracted, then row alignment and scar removal steps are carried out. For each sample, 3–5 images were taken with the clearest then chosen for publication.

Small‐angle and wide‐angle X‐ray scattering (SAXS, WAXS) experiments were performed at ambient temperature at the high‐brilliance ID2 beamline of the European Synchrotron Radiation Facility (ESRF) in Grenoble, France (Narayanan et al., 2001). Samples were prepared at the University of Edinburgh, with gels formed by cooling quiesciently at ambient room temperature. Details of the SAXS/WAXS experimental setup are given elsewhere (Bot et al., 2008). SAXS/WAXS data were collected in the ranges 0.0023 nm^−1^ < *q* < 0.25 nm^−1^, and 0.015 nm^−1^ < *q* < 1.53 nm^−1^, and 0.078 nm^−1^ < *q* < 5.7 nm^−1^, respectively, where *q* is the scattering vector defined by *q* = 4π sin*θ*/*λ* (with *θ* the scattering angle and *λ* the wavelength of the incoming X‐ray beam). These scattering vectors correspond to the inverse of the length scale being probed. Scattering data were corrected for scattering from the oil phase by subtraction of the pure oil signal.

### Computational

#### Docking calculations

The 3D models of sterols and sterol esters were generated from SMILES representation using the program OMEGA 2.5 (Hawkins et al., 2010) and Gasteiger charges were applied using AutoDockTools 1.5.6 (Morris et al., 2009). The search space was defined by a grid box centered on γ‐oryzanol compounds with 80 points of 0.375 Å spacing in each dimension. For each complex, 100 docking rounds were calculated with AutoDock 4.2 (Morris et al., 2009) using the Lamarckian genetic algorithm with the default parameters of AutoDock. The maximum number of energy evaluations was set to 5 × 10^6^, and the solvent dielectric constant was set to 2.0. The value of the solvent dielectric constant reflects the organic solvent in the oleogelating systems. The resulting docked conformations were clustered using a tolerance of 2.0 Å and the most populated cluster with the highest free energy of binding Δ*G* was selected. VMD 1.9.2 (Humphrey et al., 1996) was used to visualize the models and prepare the figures.

## RESULTS

It is very difficult to assign all the nuclear magnetic resonance (NMR) data for the synthesized compounds as they are mixtures of very similar and complex organic compounds. However, one can “absolutely” assign the removal of the ferulate to give the alcohols. In the NMR data, there is a lack of signals associated with aromatic and ester groups while the functional groups associated with the sterol are still present. The summarized ^1^H NMR is given below and full ^1^H and ^13^C spectra of starting materials and products are available in the Supporting Information S1. The melting point for the saponified γ‐oryzanol was found to be 90–100°C, while the starting γ‐oryzanol materials have the literature value of 137.5–138.5°C (Srikaeo, 2014).

NMR ^1^H (500 MHz, peak or range ppm, integral [when possible], multiplicity) 0.333 (2H, d), 0.554 (2H,d), 0.685 (1H, s), 0.781 (1H, d), 0.809 (6H, s), 0.813 (6H, s), 0.83 (1H, s), 0.846 (1H, s), 0.86 (1H,d), 0.877 (2H, s), 0.89 (n/a H, s), 0.901 (n/a H, s), 0.969 (12H, s), 1.01 (1H, s), 1.022 (3H, d), 1.037 (3H,d), 1.148–1.053 (n/a H, multiple peaks), 1.255 (n/a H, m), 1.292 (n/a H, t), 1.378–1.457 (5H, multiple peaks), 1.496 (2H, d), 1.523 (2H, d), 1.556 (n/a H, m), 1.575, (n/a H, m), 1.608 (n/a H, s), 1.624 (n/a H, m), 1.689 (3H, s), 1.752 (2H, m), 1.878 (5H, m, probably several environments), 1.997 (4H, m), 2.124 (1H, dt), 2.236 (1H, quint), 2.282 (td), 3.284 (2H, m), 3.526 (minor component, m) 3.586 (minor component, m), 4.666 (1H, d, 1 Hz), 4.716 (1H, s), 5.105 (1H, t), 5.35 (minor component, d).

**FIGURE 2:**
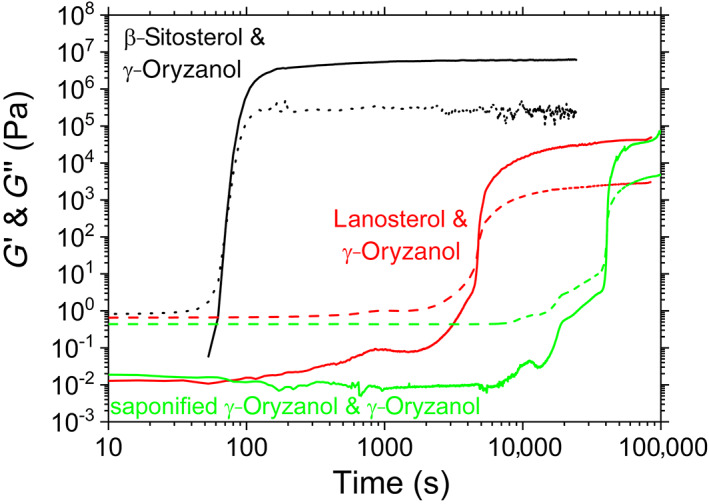
Oscillatory rheology of β‐sitosterol, lanosterol, and saponified γ‐oryzanol (major component cycloartenol) with γ‐oryzanol. Solid lines are *G*′, dashed lines are *G*″.

Rheology results for both lanosterol‐γ‐oryzanol and saponified γ‐oryzanol‐γ‐oryzanol blends dispersed in oil are shown in Figure 2, alongside data for β‐sitosterol‐γ‐oryzanol (a 10‐point moving average has been applied to the β‐sitosterol‐γ‐oryzanol data from ~200 s onwards as due to the very small displacements being measured, the extracted plateau *G*″ values are obscured by high frequency noisy otherwise). Firstly, it can clearly be seen that both novel blends do form gels. This immediately suggests that the saponification process has successfully yielded a blend of sterols capable of self‐assembly with γ‐oryzanol, and that the presence of the C30 methyl group on these sterols does not entirely prohibit gel formation. There are, however, some differences in the behavior of gels formed by β‐sitosterol‐γ‐oryzanol from those formed with lanosterol‐ γ‐oryzanol or saponified γ‐oryzanol‐ γ‐oryzanol. Firstly, although β‐sitosterol‐γ‐oryzanol mixtures have been shown to be able to allow for a certain degree of super‐cooling, the time period during which the saponified γ‐oryzanol and lanosterol system remains metastable under quiescent conditions or with mild agitation without gelling is two orders of magnitude longer. Once this quiescent period is over, the gelation process is much slower, and the final gels are weaker. The duration of this quiescent period and degree to which the gels are weaker is orders of magnitude different from that observed not only for β‐sitosterol γ‐oryzanol blends, but for blends of other sterols such as cholestanol, cholesterol, and stigmasterol (Dalkas et al., 2018). This suggests that there may be a significant free energy barrier to gelation in this system, the intermolecular bonding may be weaker, or that tubules are present but are not forming a percolating network. This is the first indication that the superficially slight differences in the sterol structures are having significant effects on the gel.

**FIGURE 3:**
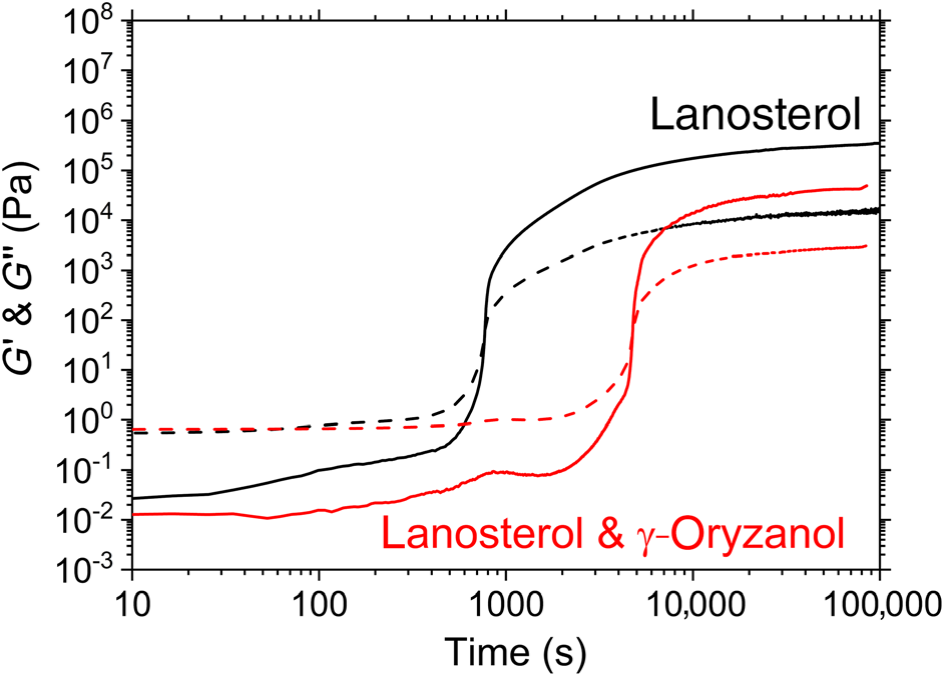
Oscillatory rheology of lanosterol, and of lanosterol blended with γ‐oryzanol. Solid line is *G*′ dashed line is *G*″.

Interestingly, rheology also reveals that lanosterol will form a gel *without* the addition of γ‐oryzanol (Figure 3). The ability of lanosterol to form gels in organic solvents has been identified before (Li et al., 2006), but not in sunflower oil. Figure 3 shows that the gels formed with 4% lanosterol and no γ‐oryzanol (black line) make firmer gels than those with 4% lanosterol blended with 6% γ‐oryzanol (red line), that is, adding γ‐oryzanol actually weakens the gel.

**FIGURE 4:**
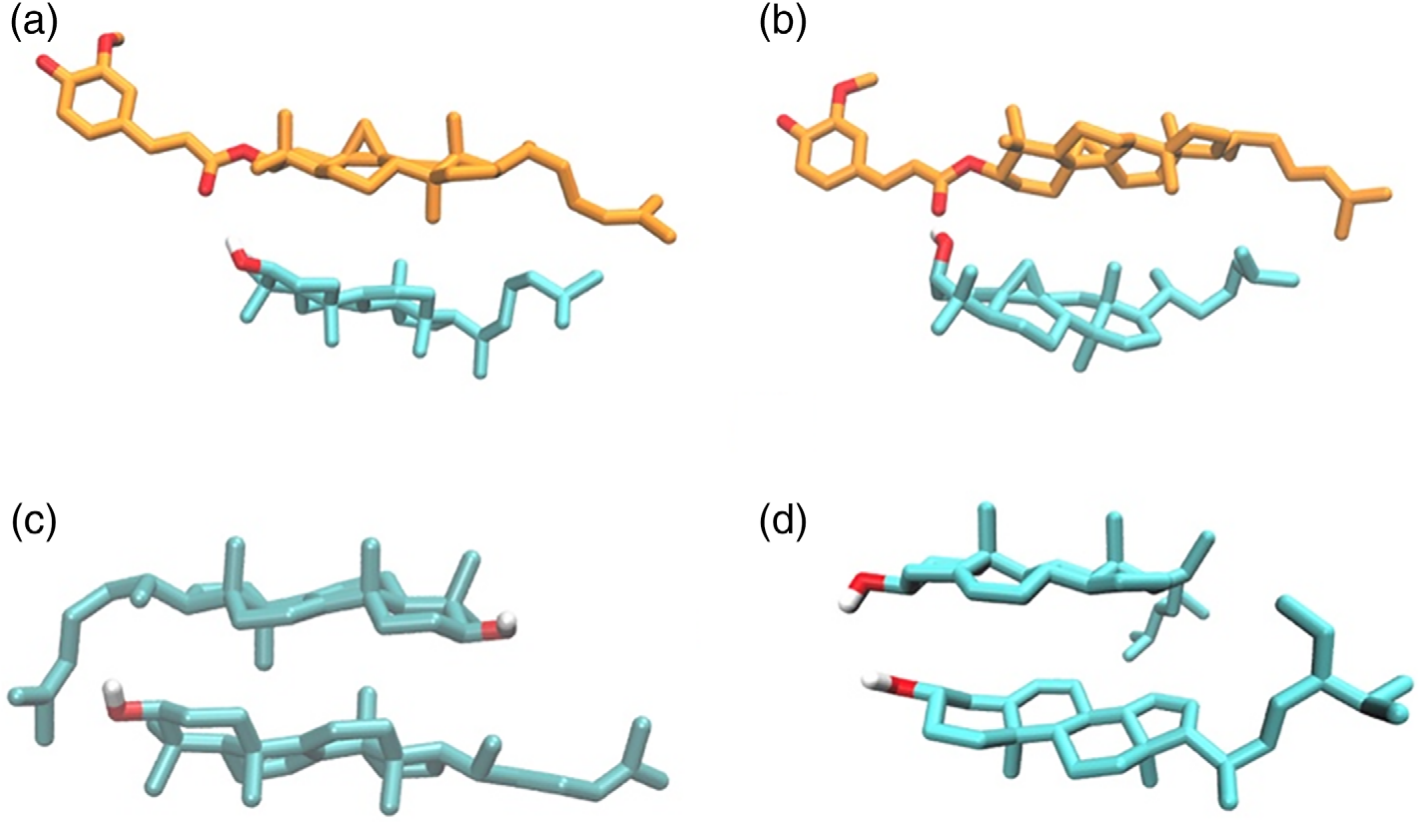
Docked conformations of (a) γ‐oryzanol‐lanosterol, (b) γ‐oryzanol‐cycloartenol, (c) lanosterol‐lanosterol, (d) β‐sitosterol‐β‐sitosterol dimers. γ‐Oryzanol is shown with orange carbons, lanosterol, cycloartenol, and β‐sitosterol with cyan carbons.

To understand how molecular structure influences the gel formation and subsequent structure in the lanosterol, lanosterol‐γ‐oryzanol, and saponified γ‐oryzanol‐γ‐oryzanol systems, docking simulations were employed, as they have proven to be an instructive means of understanding these systems in the past. Docking simulations were initially carried out for lanosterol with γ‐oryzanol (Figure 4a), and cycloartenol (which should be the main component of saponified γ‐oryzanol) with γ‐oryzanol (Figure 4b). For both systems, docking results revealed a similar binding pose to that previously observed as the top‐ranked docking conformation model of γ‐oryzanol and β‐sitosterol (Dalkas et al., 2018). This is consistent with the fact gel formation occurs in these systems, unlike in blends of, for example, β‐sitosterol with cholesteryl hemisuccinate where the binding pose differs significantly and gels do not form (Dalkas et al., 2018). Docking simulations can also reveal the dominant molecular interactions in the pure lanosterol organogel. In the system where two lanosterol molecules were docked, the cluster with the lowest mean free energy of binding exhibited an antiparallel head‐to‐tail conformation (Figure 4c). On the other hand, because of the absence of the C30 methyl group in β‐sitosterol, the sterane groups of β‐sitosterol dimers were docked in a parallel “head‐to‐head, tail‐to‐tail” manner and the wedge shape was not observed for the top‐ranked docking conformation. The presence of this wedge confirmation in the lanosterol‐lanosterol dimer versus its absence in the β‐sitosterol‐β‐sitosterol dimer gives a clear indication as to why pure lanosterol may form gels without γ‐oryzanol whereas β‐sitosterol will not.

**FIGURE 5:**
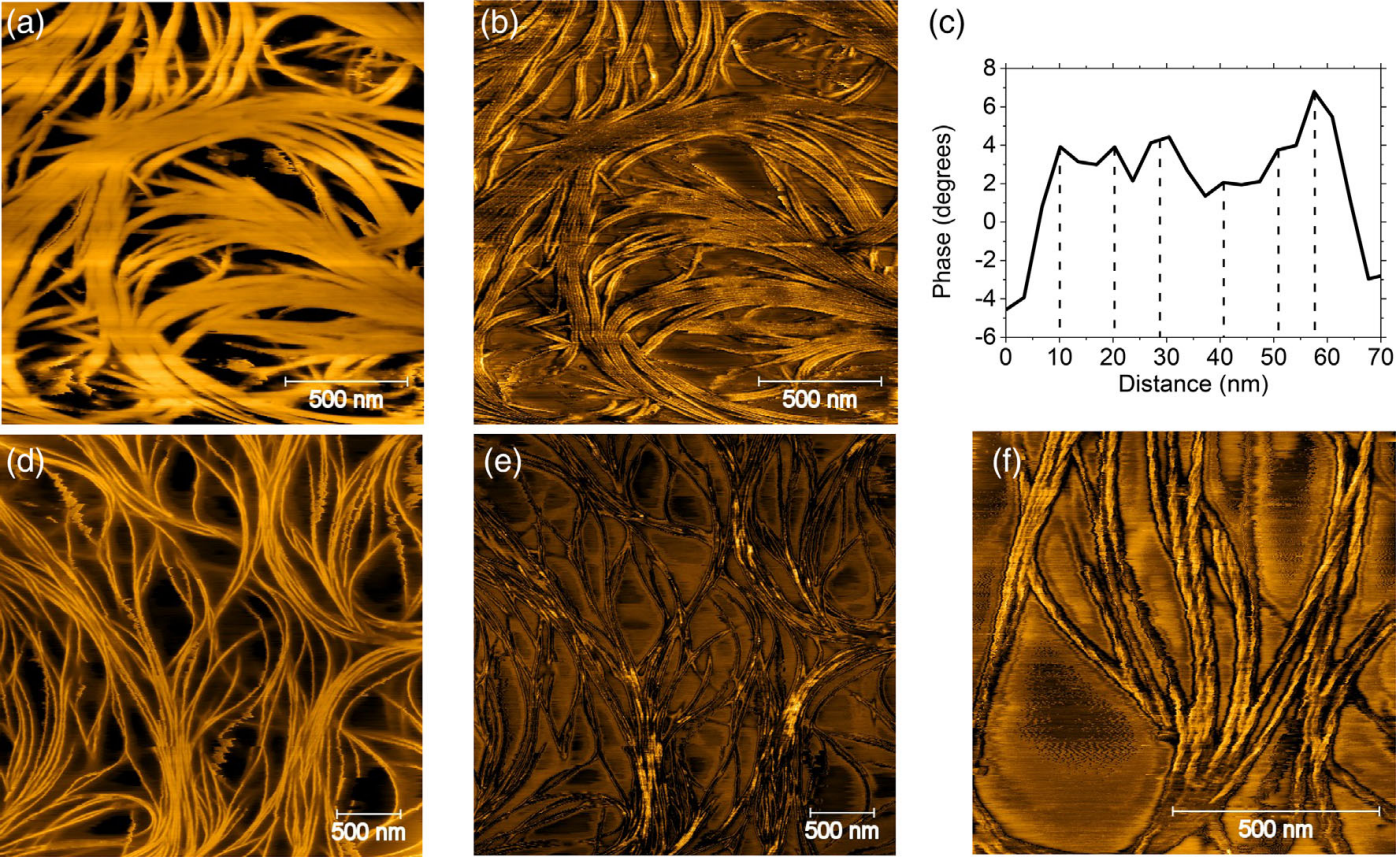
(a) AFM height and (b) AFM phase images of a saponified γ‐oryzanol‐γ‐oryzanol gel, (c) a profile taken through one of the fibrous ribbons shown in (b); (d) AFM height image and (e) AFM phase image of a lanosterol‐γ‐oryzanol gel; (f) AFM phase image of a lanosterol gel, without γ‐oryzanol

To better understand the gel structure, the gel network morphology can be directly imaged using AFM. In Figure 5a,b, the saponified γ‐oryzanol‐γ‐oryzanol gel shows the same dense mat of helical ribbons that were previously reported for β‐sitosterol‐γ‐oryzanol gels (see Supporting information S1 for an example image) (Matheson et al., 2017). From the phase image in Figure 5b, one can resolve the structure of individual tubules of saponified γ‐oryzanol and γ‐oryzanol which are ~10 nm in diameter (see profile in Figure 5c), and therefore are of the same size as those observed in other sterol‐γ‐oryzanol gels (Dalkas et al., 2018). For lanosterol‐γ‐oryzanol gels a fibrous structure is also revealed (Figure 5d,e), however, the fibers do not seem to form a dense mat of ribbons, and the fibrous sub‐structure of 10 nm tubules within the larger bundles is not visible.

For lanosterol only gels, the phase image presented in Figure 5f shows that the gel is fibrous, but again, rather than featuring ribbons of 10 nm tubules, it shows a system of twisted fibers. Considering the molecular docking simulations presented earlier in Figure 4, it is unsurprising that the structure of the lanosterol gel is different given that it is presumably the consequence of an entirely different stacking motif, that is, head to tail with a wedge cross‐section, and without the hanging ferulic acid groups which are believed to encourage parallel adhesion of tubules into ribbons (Dalkas et al., 2018; Matheson et al., 2017).

**FIGURE 6:**
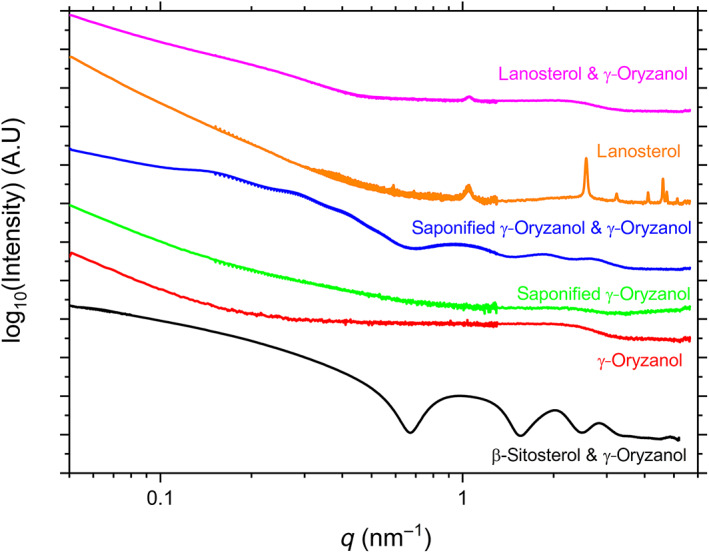
SAXS results for sterols and sterol‐esters suspended in sunflower oil obtained at room temperature. Black is from β‐sitosterol blended with γ‐oryzanol, red is γ‐oryzanol, green is the saponified γ‐oryzanol, blue the saponified γ‐oryzanol blended with γ‐oryzanol, orange is lanosterol, and pink is lanosterol blended with γ‐oryzanol.

Whereas molecular docking simulations reveal much about the molecular packing of the dimers, and AFM reveals much about the overall microstructure of the gels, small angle x‐ray scattering measurements (SAXS), have proven to be a highly effective tool in probing the intermediate length scale of the system—the structure of the individual fibers and tubules. For instance, it was instrumental in revealing that the fibers apparent in sterol‐sterol ester oleogels are hollow tubules, and it gives information on the internal structure of any fibers, which may not be probed by AFM (den Adel et al., 2010; Sawalha et al., 2015). The results are shown in Figure 6 and can be summarized as follows. The black trace is data for β‐sitosterol blended with γ‐oryzanol. Similar to as has been previously documented (Bot et al. 2008, 2009), it shows peaks at 0.98, 2.05, and 2.83 nm^−1^. This scattering pattern has previously been assigned to a hollow tubule. For blends of saponified γ‐oryzanol and γ‐oryzanol (blue trace), a scattering pattern very similar to that for β‐sitosterol blended with γ‐oryzanol is observed, with a peak at 0.95 nm^−1^, then successively weaker peaks at 1.85 and 2.69 nm^−1^, again suggesting a hollow tubule of very similar dimensions. The only significant difference observed here for saponified γ‐oryzanol and γ‐oryzanol is that there are additional peaks in the 0.1–1 nm^−1^ region not apparent in β‐sitosterol blended with γ‐oryzanol, the most likely cause for these are the highly ordered laterally aligned tubule domains observed in Figure 5. For lanosterol blended with γ‐oryzanol (pink trace), the tell‐tale features associated with hollow tubule formation in the 1–10 nm^−1^ region are absent, giving a strong indication that tubules are not forming in the same manner. Instead, the main feature is a single sharp peak at 1.05 nm^−1^, and (in common with the other systems) at low *q* values there are largely featureless slopes, which most likely correspond to the aggregation of fibers observed in AFM images. This 1.05 nm^−1^ peak also appears in the scattering pattern of lanosterol without γ‐oryzanol present (orange trace), alongside several additional peaks at higher wavenumbers which are absent when blended with γ‐oryzanol. This would seem to imply that molecular packing in the lanosterol‐γ‐oryzanol system may be more similar to that of the pure lanosterol gels than the β‐sitosterol or saponified γ‐oryzanol blended with γ‐oryzanol, and would give further confirmation that lanosterol‐γ‐oryzanol gels are not forming in the same manner as other sterol‐γ‐oryzanol blends.

These results are entirely consistent with the structures revealed in the AFM images, that is, the gelation process in saponified γ‐oryzanol‐γ‐oryzanol gels is due to the formation and entanglement of hollow tubules of approximately 10 nm diameter, with both AFM images and scattering data closely matching that previously observed for β‐sitosterol blended with γ‐oryzanol. In pure lanosterol or lanosterol‐γ‐oryzanol gels, individual 10 nm tubules are not observed in either AFM images or SAXS, which would strongly suggest a different sort of fiber nanostructure and formation process but the precise structure of these fibers cannot be stated with confidence.

## CONCLUSIONS

To summarize, saponified γ‐oryzanol behaves in a very similar manner to other sterols previously investigated (Dalkas et al., 2018). It forms hollow tubules when blended with γ‐oryzanol, which stick together as ribbons and enmesh to form gels. The resultant gels form more slowly and are substantially weaker than the equivalent gels formed with β‐sitosterol or other previously investigated sterol esters. Given that the SAXS and AFM data is so similar, it would suggest that the cause of this weakness is not due to a significantly different gel structure or formation process, but due to differences in either the strength of the intermolecular bonding which holds the tubules together or the inter‐tubular interactions which cause the tubules to adhere to one another. Lanosterol, however, behaves significantly differently. Unlike other sterols, it can form gels in sunflower oil without the addition of γ‐oryzanol. The mechanism for this seems to be that the wedge‐shaped head‐to‐tail stacking confirmation (unlike the head‐to‐head confirmation exhibited in β‐sitosterol dimers) it exhibits allows it to form thick, twisted fibers unlike the ribbons of tubules seen in β‐sitosterol‐γ‐oryzanol blends. Blends of lanosterol and γ‐oryzanol appear to be an interesting intermediate case—this blend forms a gel, and molecular docking simulations suggest that lanosterol and γ‐oryzanol form a stable dimer with a very similar confirmation to that of a β‐sitosterol‐γ‐oryzanol dimer. However, neither the AFM nor SAXS data suggest that this system contains the hollow tubules typical of the β‐sitosterol‐γ‐oryzanol system, instead exhibiting features, which share more in common with the pure lanosterol gel. This would seem to suggest that the tendency for lanosterol to aggregate into twisted fibers dominates over the tendency for lanosterol‐γ‐oryzanol to self‐assemble into tubules. Furthermore, the fact that lanosterol gels weaken on the addition of γ‐oryzanol would suggest that γ‐oryzanol may disrupt the structure of lanosterol stacking. These results show that very minor changes to the steroidal core and acyclic tail of the sterol, such as the presence of the 30 methyl group, can have a very significant effect on gel formation, highlighting the complexity of oleo‐gelator selection and design, and contribute to the growing collection of design principles for the synthesis of novel sterols and sterol‐esters for use as organogelators.

## AUTHOR CONTRIBUTIONS

Andrew B. Matheson, Paul S. Clegg, Gareth O. Lloyd, Georgios Dalkas, Stephen R. Euston devised the study. Gareth O. Lloyd and Aaliyah Hart synthesized and characterized the raw materials. Andrew B. Matheson performed gel preparation, rheology, and AFM measurements. Georgios Dalkas performed molecular simulations. Vasileios Koutsos assisted in analyzing AFM measurements. Arjen Bot and Ruud den Adel performed scattering experiments and associated analysis. Andrew B. Matheson led the writing of the manuscript, all other authors contributed to the writing and revisions of the manuscript.

## CONFLICT OF INTEREST

The authors declare that they have no conflict of interest.

## ETHICS STATEMENT

No human or animal studies were performed as part of this work.

## Supporting information


**FIGURE S1** ^1^H (top) and ^13^C (bottom) of γ‐oryzanol. Peaks in the 6–8 ppm range of the ^1^H NMR and the peaks 150–170 ppm range of the ^13^C NMR are associated with the ester furelate.
**FIGURE S2**: ^1^H NMR of the saponified γ‐oryzanol. Top shows no aromatic signals associated with the ferulate functionality. Blow ups of the data to show the different signals of the compound mixture.
**FIGURE S3**: ^13^C NMR of the saponified γ‐oryzanol. No clear signal associated with the aromatic or carbonyl peaks indicate the ester has been removed.
**FIGURE S4**: An AFM height image of a β‐sitosterol‐γ‐oryzanol gel.
