# Supplementary material for: Exploring how changes to the steroidal core alter oleogelation capability in sterol: γ‐oryzanol blends
Source: J Am Oil Chem Soc. 2022 Jul 29;99(11):943–50. doi: 10.1002/aocs.12624 (PMC9796822; doi:10.1002/aocs.12624)
Supplement: Supplementary file 1 — FIGURE S1 1H (top) and 13C (bottom) of γ‐oryzanol. Peaks in the 6–8 ppm range of the 1H NMR and the peaks 150–170 ppm range of the 13C NMR are associated with the ester furelate. FIGURE S2: 1H NMR of the saponified γ‐oryzanol. Top shows no aromatic signals associated with the ferulate functionality. Blow ups of the data to show the different signals of the compound mixture. FIGURE S3: 13C NMR of the saponified γ‐oryzanol. No clear signal associated with the aromatic or carbonyl peaks indicate the ester has been removed. FIGURE S4: An AFM height image of a β‐sitosterol‐γ‐oryzanol gel. [file AOCS-99-943-s001.docx]

# Exploring how changes to the steroidal core alter oleogelation capability in sterol – γ-oryzanol blends –Supplementary Information

Andrew B. Matheson *1, Georgios Dalkas 2, Gareth O. Lloyd 3, Aaliyah Hart 3, Arjen Bot 4,5 , Ruud den Adel 4, Vasileios Koutsos 6, Paul S. Clegg 1, Stephen R. Euston 2,

1. School of Physics and Astronomy, University of Edinburgh, James Clerk Maxwell Building, Edinburgh, EH9 3FD, UK,
2. School of Engineering and Physical Sciences, Institute of Biological Chemistry, Biophysics and Bioengineering, Heriot-Watt University, Edinburgh, EH14 4AS, UK,
3. School of Chemistry, Joseph Banks Laboratories, University of Lincoln, Lincoln, LN6 7TS, UK
4. Unilever Foods Innovation Centre, Bronland 14, NL-6708 WH Wageningen, The Netherlands
5. Laboratory of Physics and Physical Chemistry of Foods, Department of Agrotechnology and Food Sciences, Wageningen University and Research, Bornse Weilanden 9, NL-6708 WG Wageningen, The Netherlands
6. School of Engineering, Institute for Materials and Processes, The University of Edinburgh, Sanderson Building, King’s Buildings, Edinburgh EH9 3FB, United Kingdom

* corresponding author email: a.matheson@ed.ac.uk

# NMR

Spectra was collected on a Bruker 500 MHz NMR. Compounds were dissolved in CDCl3, with the solvent peak acting as the reference value.

## γ-oryzanol NMR


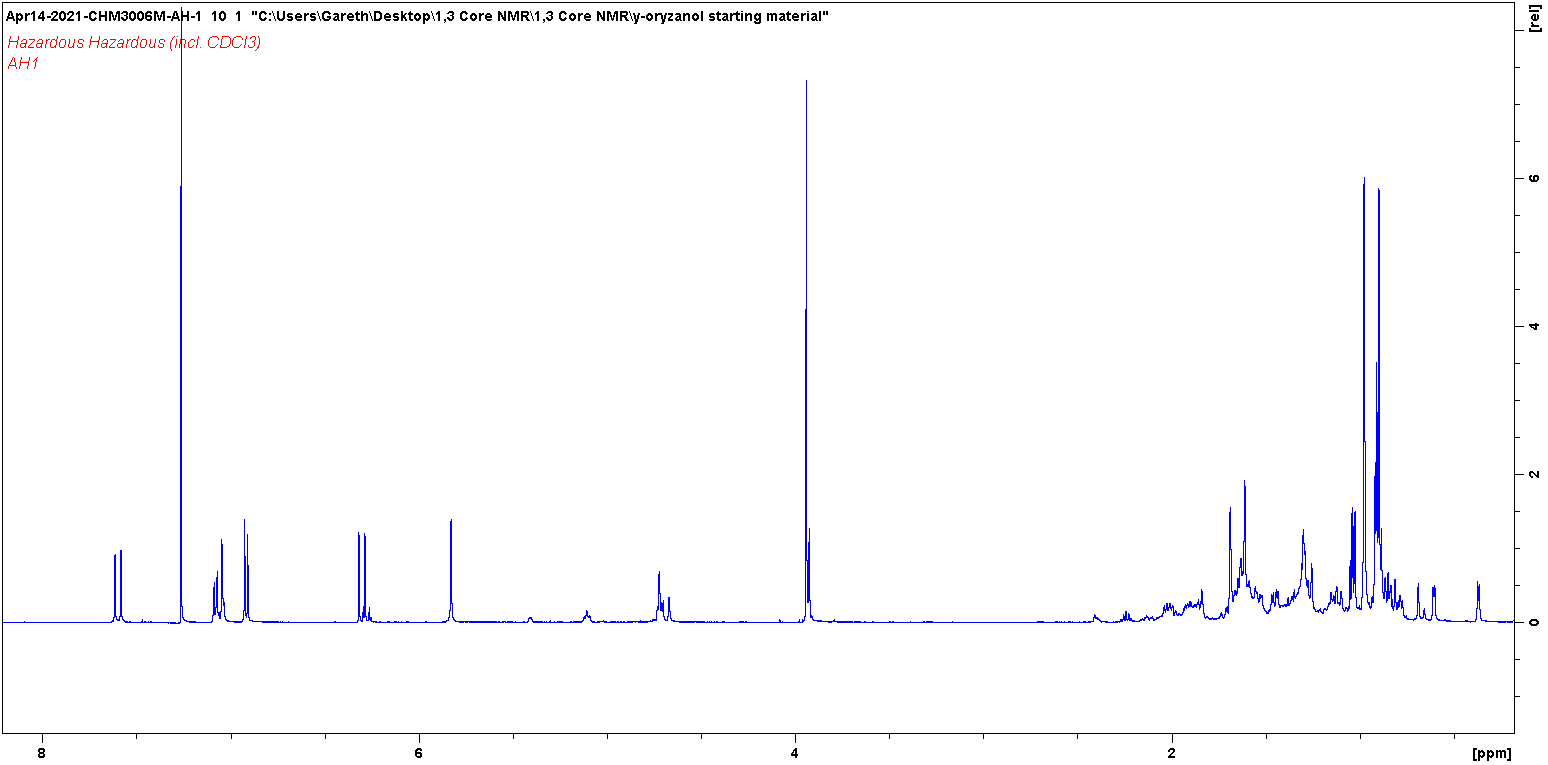


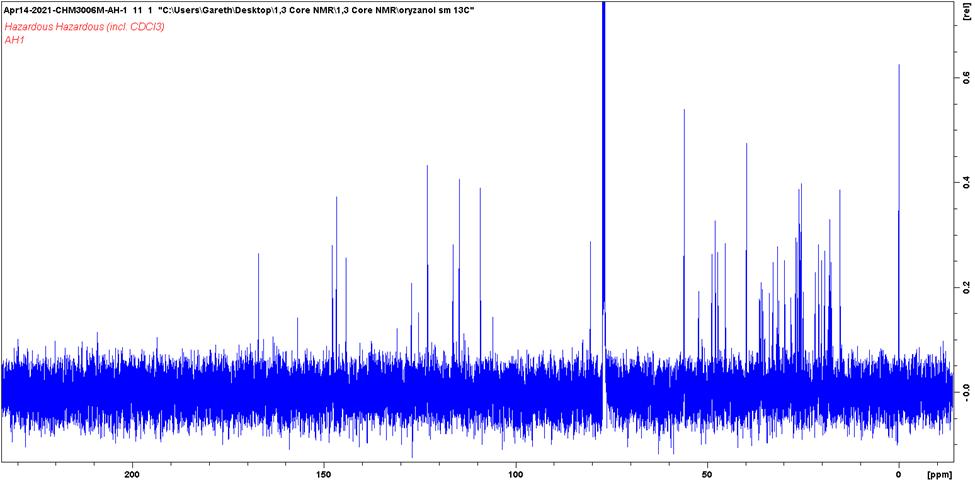


**Figure S1.** ^1^H (top) and ^13^C (bottom) of **γ-oryzanol**. Peaks in the 6-8 ppm range of the ^1^H NMR and the peaks 150-170 ppm range of the ^13^C NMR are associated with the ester furelate.

## Saponified γ-oryzanol NMR


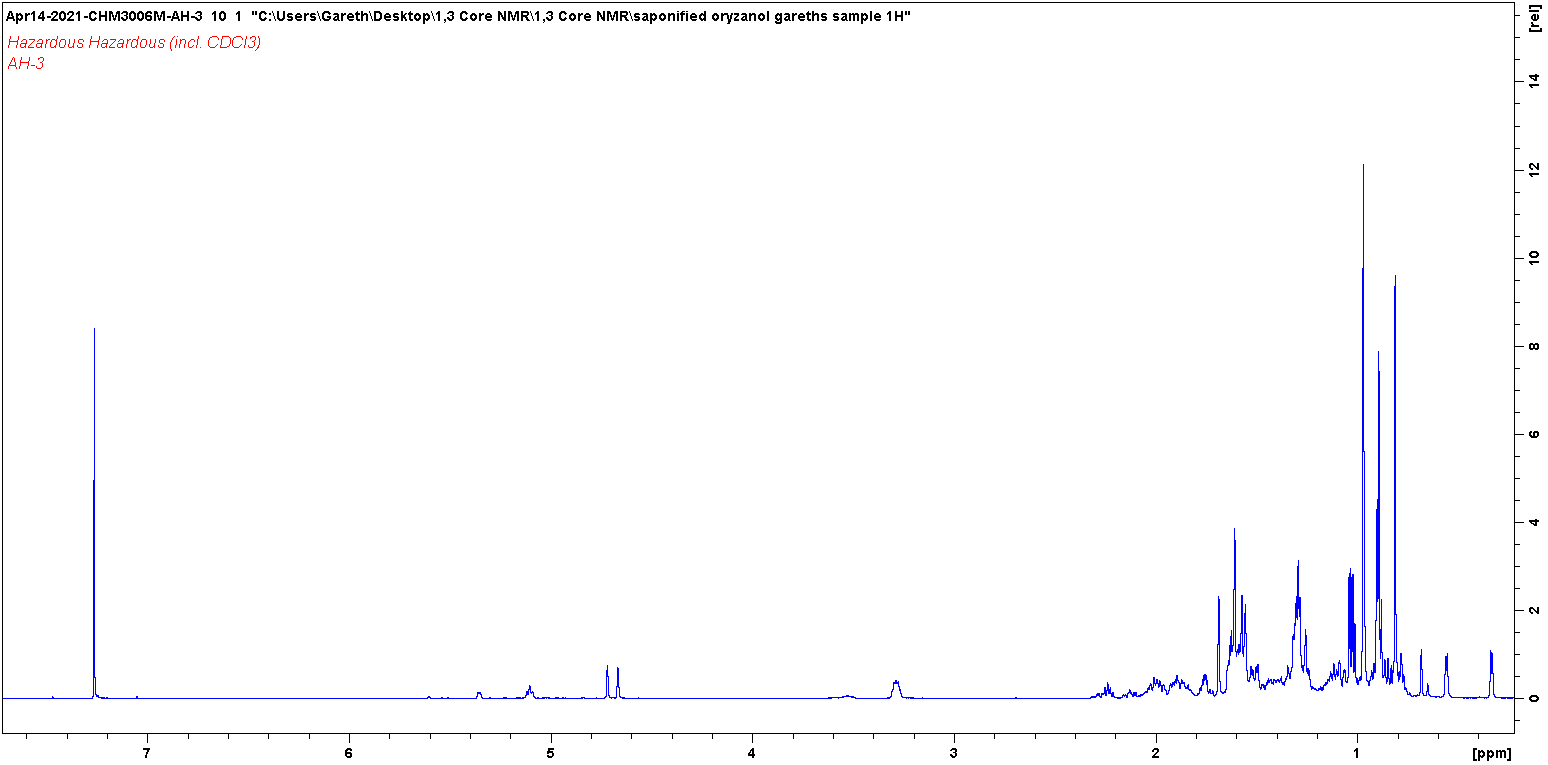


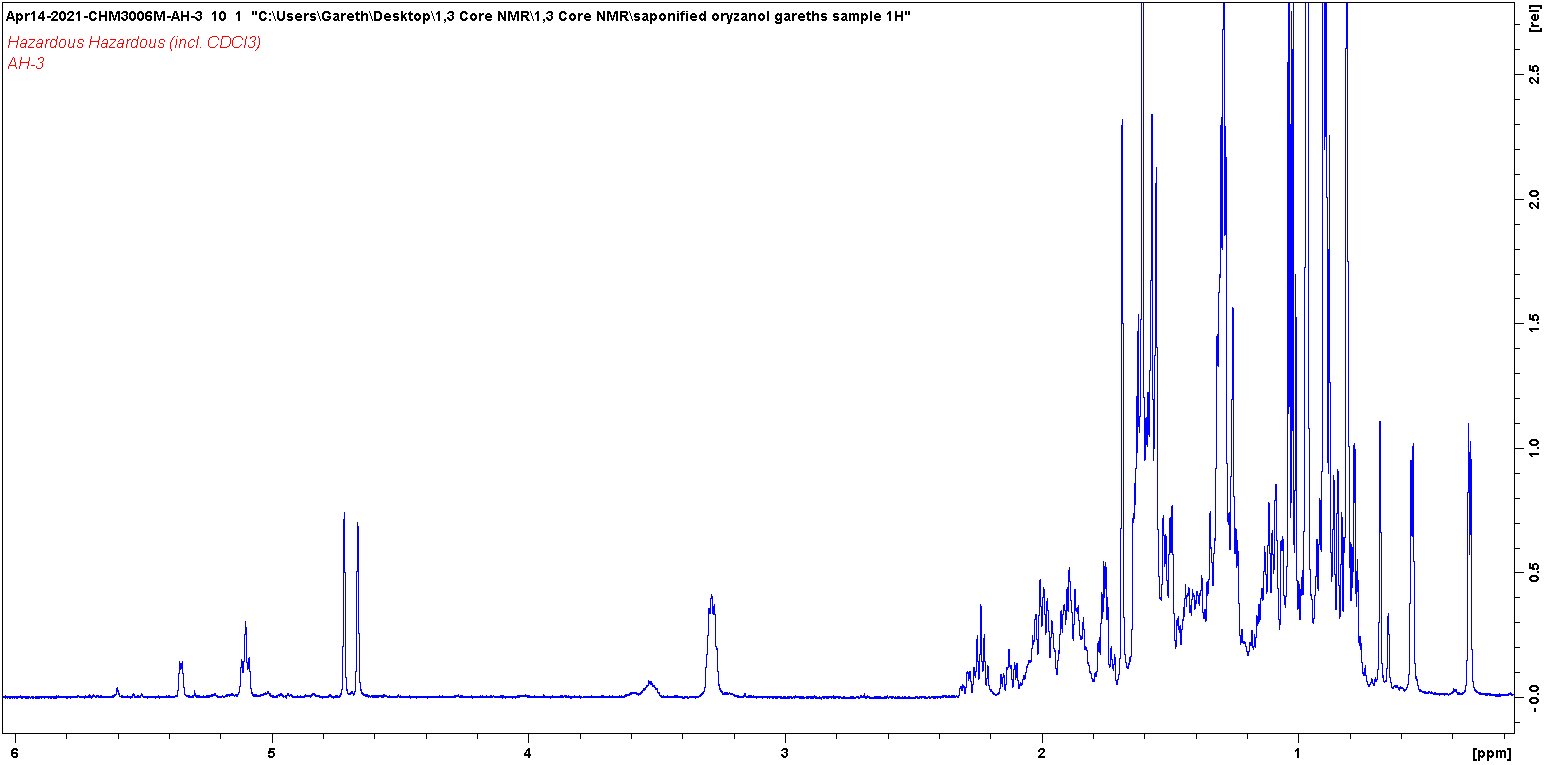


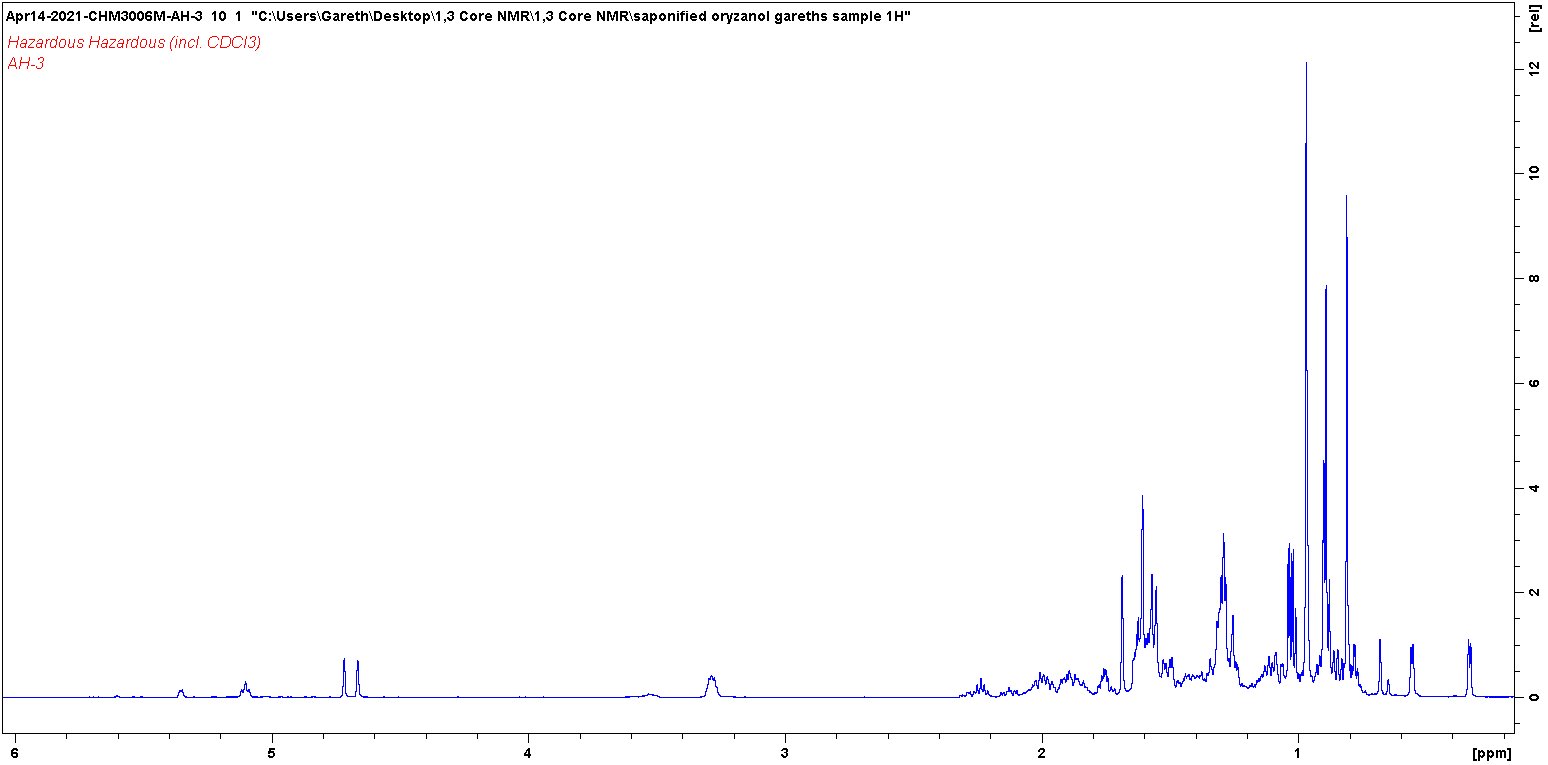


**Figure S2.** ^1^H NMR of the saponified **γ-oryzanol**. Top shows no aromatic signals associated with the ferulate functionality. Blow ups of the data to show the different signals of the compound mixture.


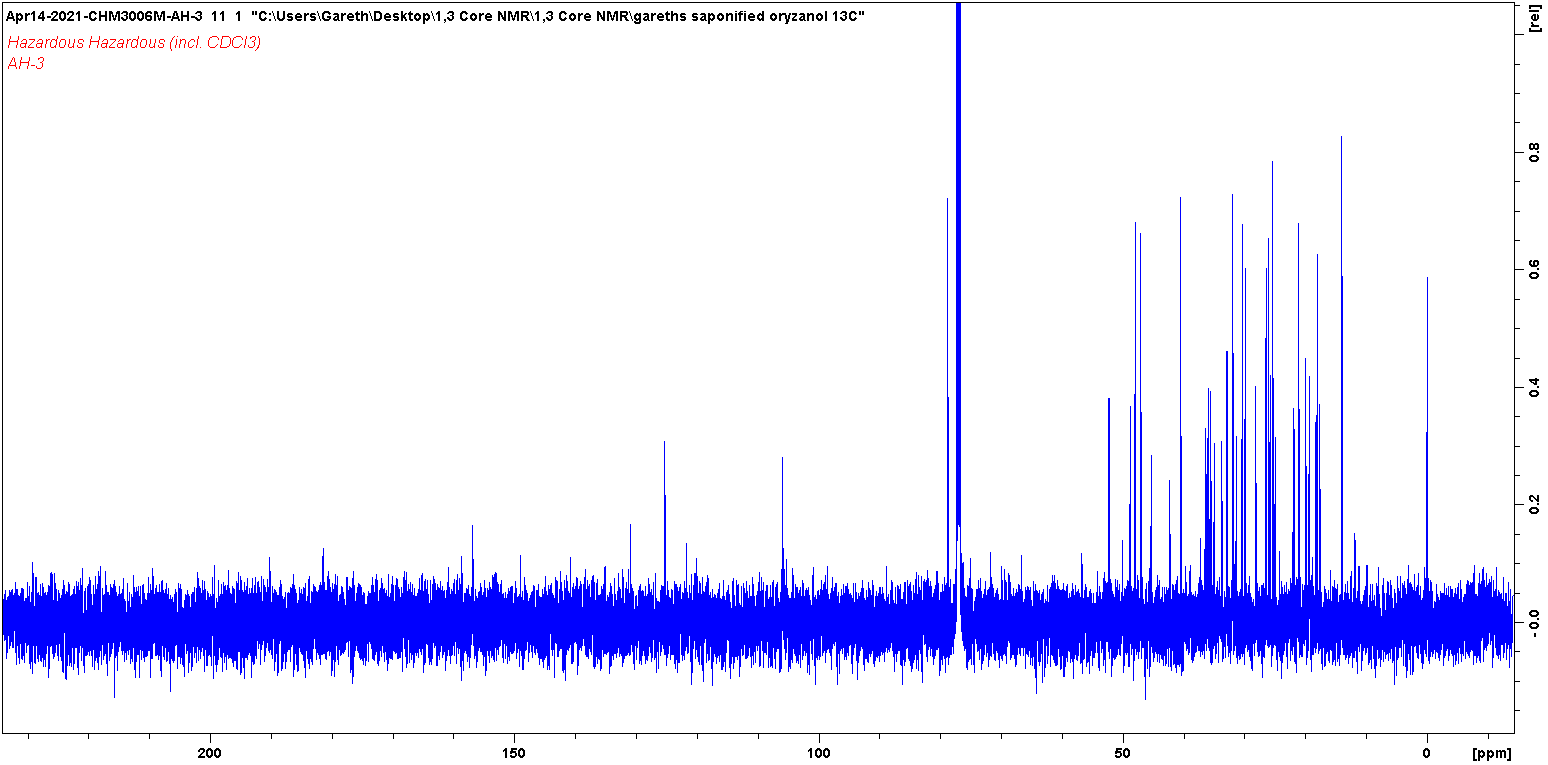


**Figure S3.** ^13^C NMR of the saponified **γ-oryzanol**. No clear signal associated with the aromatic or carbonyl peaks indicate the ester has been removed.

# AFM


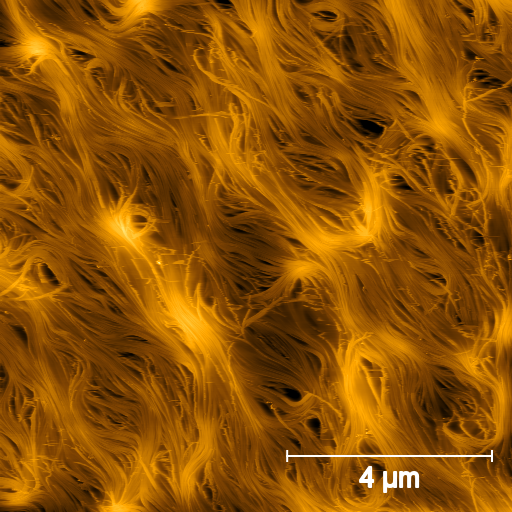


**Figure S4** – An AFM height image of a β - sitosterol - γ-oryzanol gel.
